# Food cue reactivity in successful laparoscopic gastric banding: A sham-deflation-controlled pilot study

**DOI:** 10.3389/fnhum.2022.902192

**Published:** 2022-08-25

**Authors:** Marinka M. G. Koenis, Janet Ng, Beth Anderson, Michael C. Stevens, Darren S. Tishler, Pavlos K. Papasavas, Andrea Stone, Tara McLaughlin, Allison Verhaak, Mirjana J. Domakonda, Godfrey D. Pearlson

**Affiliations:** ^1^Olin Neuropsychiatry Research Center, Institute of Living at Hartford Hospital, Hartford, CT, United States; ^2^Department of Psychiatry, Yale University School of Medicine, New Haven, CT, United States; ^3^Division of Metabolic and Bariatric Surgery, Hartford Hospital, Hartford, CT, United States; ^4^Department of Neuroscience, Yale University School of Medicine, New Haven, CT, United States

**Keywords:** fMRI, obesity, LAGB, laparoscopic adjustable gastric banding, food cue, bariatric (weight loss) surgery, lap-band, brain

## Abstract

Laparoscopic adjustable gastric banding (LAGB) offers a unique opportunity to examine the underlying neuronal mechanisms of surgically assisted weight loss due to its instant, non-invasive, adjustable nature. Six participants with stable excess weight loss (%EWL ≥ 45) completed 2 days of fMRI scanning 1.5–5 years after LAGB surgery. In a within-subject randomized sham-controlled design, participants underwent (sham) removal of ∼ 50% of the band’s fluid. Compared to sham-deflation (i.e., normal band constriction) of the band, in the deflation condition (i.e., decreasing restriction) participants showed significantly lower activation in the anterior (para)cingulate, angular gyrus, lateral occipital cortex, and frontal cortex in response to food images (*p* < 0.05, whole brain TFCE-based FWE corrected). Higher activation in the deflation condition was seen in the fusiform gyrus, inferior temporal gyrus, lingual gyrus, lateral occipital cortex. The findings of this within-subject randomized controlled pilot study suggest that constriction of the stomach through LAGB may indirectly alter brain activation in response to food cues. These neuronal changes may underlie changes in food craving and food preference that support sustained post-surgical weight-loss. Despite the small sample size, this is in agreement with and adds to the growing literature of post-bariatric surgery changes in behavior and control regions.

## Introduction

Although laparoscopic gastric banding (LAGB) is a safe procedure, its popularity with patients and surgeons has declined in recent years due to its relative lack of effectiveness in producing optimal weight loss and the high rate of band revisions (Khoraki et al., 2018). However, LAGB presents a unique opportunity to examine the potential underlying neuronal mechanisms of surgically assisted weight loss, as the band is instantly adjustable in a non-invasive manner.

Many factors are involved in appetite regulation, including vagal mechanoreceptors responsible for signaling satiation, hormones such as ghrelin and leptin, environmental cues, learned behaviors, and genes associated with reward, learning, and cognitive control (Andermann and Lowell, 2017). Previous studies have shown that healthy weight individuals activate several brain regions associated with sensory processing and reward during food viewing tasks (van der Laan et al., 2011; Tang et al., 2012; van Meer et al., 2015). Activity in these regions is increased when subjects are in a fasted compared to fed state (LaBar et al., 2001; Goldstone et al., 2009; Siep et al., 2009), but is decreased when subjects are administered peptide YY and glucagon-like peptide-1 (De Silva et al., 2011), which mimic satiety. Compared to healthy weight individuals, individuals with obesity demonstrate stronger activity in these regions in response to food images (Martin et al., 2010; Scharmüller et al., 2012; Brooks et al., 2013; Luo et al., 2013; Pursey et al., 2014), and decreased activation after bariatric surgery and behavioral weight loss interventions (Bruce et al., 2012; Murdaugh et al., 2012; Nock et al., 2012; Ochner et al., 2012a,b, 2011; Faulconbridge et al., 2016; Holsen et al., 2018). Together, these studies suggest a role for neuronal activation patterns in weight loss.

The main mechanism of LAGB is thought to be activation of the peripheral satiety system without necessarily restricting meal size (Burton and Brown, 2011). Two studies have examined neuronal responsivity to food cues in relation to LAGB (Bruce et al., 2012; Ness et al., 2014). Bruce and colleagues found that, compared to pre-operative neuroimaging data, LAGB participants showed decreased neuronal activation in brain regions related to food motivation and reward (medial prefrontal and insular cortices, parahippocampal gyrus), and increased activation in regions involved in cognitive control and inhibition (prefrontal cortex) (Bruce et al., 2012). Ness et al. (2014) reported that LAGB participants with higher pre-operative activity in brain regions associated with cognitive control (prefrontal cortex, posterior cingulate) showed more weight loss 3–6 months post-surgery. The brain’s response to food pictures is also changed after Roux-en-Y gastric bypass (RYGB) and sleeve gastrectomy (SG) (Pursey et al., 2014; Faulconbridge et al., 2016; Zoon et al., 2018; Baboumian et al., 2019; Bach et al., 2021).

Together, these studies suggest that LAGB may influence neuronal activation in reward and cognitive control circuits, and further imply that individual differences in baseline brain activation may impact weight-loss success in the post-operative period. No studies have been performed to assess the effect that the degree of mechanical pressure applied to the proximal stomach by the band has on brain activation. Here we utilize the adjustable nature of the gastric band to investigate the impact of acute loss of stomach restriction on neuronal activation during food viewing. This study may further elucidate mechanisms underlying post-surgical weight loss, post-surgical changes in brain activation, and the relation between post-surgical neuronal changes associated with baroreceptor activation in the proximal gastrointestinal system.

## Methods

### Participants

Out of 14 enrolled adult females, 12 were included, and 6 had MRI data available to identify neuronal mechanisms associated with responses to food cues and their association with partial band deflation (see Table 1 for demographics). Exclusion was due to: MRI contraindication (*n* = 1); did not reach 45% EWL (*n* = 1); did not show up for the second scan (*n* = 2); technical error during scan (*n* = 1); too much movement (*n* = 1, > 35% volumes with framewise displacement > 0.5 mm); an older LAGB version which uses different mechanism (less fluid), possibly leading to a different restriction-difference when removing 50% of the fluid (*n* = 1); scanned with different scan parameters (*n* = 1). Inclusion criteria were: LAGB (LAP−BAND, Allergan, Santa Barbara, CA, United States) at least 1 year prior to study enrollment in 2013 and 2014; stable weight loss with an optimally adjusted band (the “green zone:” patient experiences early satiety following meals and prolonged satiety with reduced appetite even after long periods between eating); percent excess weight loss (%EWL) ≥ 45. The study was approved by the Hartford Hospital institutional review board. All participants provided written informed consent.

**TABLE 1:** Participant information [mean (SD; range)], all women (*n* = 6).

Age	39.7(9.3;27−51)
BMI	28.3(3.4;25.9−34.4)
Pre-surgery BMI	42.3(5.2;36−51)
% EWL	80.8(13.6;58.5−93.9)
Time surgery to 1st scan (years)	3.5(1.5;1.6−5.1)
Time between scans (days)	10.0(12.5;1−35)
Fluid removed (cc)	3.3(0.8;3.0−5.0)

EWL, Excess Weight Loss.

### Procedure

To investigate the neuronal relations of mechanical restriction, we used a within-subject design where we compare a real deflation to a sham-deflation. We used a single-blind controlled design to avoid any possible influence of the cognitive knowledge of the condition. We also randomly assigned participants to the sham or real deflation to avoid a “learning” or “training” effect which could occur when, e.g., deflation scan is always after a “no-deflation” scan. Thus, this study followed a within-subject randomized single-blind sham-controlled design.

Magnetic Resonance Images (MRI) were acquired from a Siemens Skyra 3T scanner (Siemens, Erlangen, Germany) at the Olin Neuropsychiatry Research Center, Institute of Living, Hartford Hospital. Participants underwent MRI scanning after an overnight fast on two separate days ranging 1–35 days apart. Time of scanning (08:00–09:00 a.m.) was the same for all participants and both sessions to minimize time-of-day confounds. Deflation and sham-deflation procedures were performed by a bariatric surgeon or physician assistant at the clinic. For the deflation session, 50% of the fluid was removed from the individual’s band; for the sham-deflation session, participants’ ports were accessed, fluid was removed and then immediately replaced, without net volume change. Participants were blinded to condition and deflation/sham-deflation days were assigned at random and counterbalanced for all participants. Brain activity in response to food images was measured through the food cue reactivity task (Goldstone et al., 2009) administered approximately 30 min after deflation/sham-deflation to allow for transportation to the research center and administration of a visual analog scale (VAS) on food intake motivation (Flint et al., 2000). After MRI-scanning, participants were asked to guess their band’s condition (deflated vs. no change). Back at the clinic, those in the deflation condition had their fluid volume restored; those in the sham-deflation condition had their port accessed without actual fluid replacement.

### Food cue reactivity task

The Food Cue Reactivity Task was modified in-house from the Alcohol Cue Reactivity Task (Dager et al., 2013). It consisted of 44 food images [22 high energy-dense foods (HED), e.g., ice cream, cookies; 22 low energy-dense foods (LED), e.g., salad, fruit] matched on valence, arousal, image complexity, brightness, and hue, and 44 degraded images to serve as a visual baseline. Objective values of image properties were obtained with a photo editing program (GIMP, Berkeley, CA). Matching was confirmed by employee ratings. Degraded images were created from the food images using Image Shuffle (San Diego, CA). To improve signal in the primary task condition and contrast of interest, food images were presented twice each; degraded images were presented once. Each picture was presented for 1,750 ms followed by a fixation cross presented for 250–4,250 ms. Participants were asked to indicate whether they “liked,” “disliked,” or felt “neutral” about each image by pressing a corresponding button within 2,000 ms of image presentation; ratings and reaction times were logged via a fiber-optic response box. Total duration of the task was 5:54 min, and included an initial 9 s fixation period to allow for magnetization stabilization (excluded from analyses). Prior to the scan session, participants practiced the task outside the scanner using non-food pictures and a computer keyboard.

### Image acquisition, processing, and analyses

Whole-brain T1-weighted structural images were acquired with five sagittally-collected magnetization-prepared rapid gradient-echo (MPRAGE) scans with the following parameters: TR/TE/TI = 2,200/2.88/794 ms, flip angle = 13º, 0.8 mm isotropic voxels. Whole-brain T2-weighted functional images were collected in the axial plane with an echoplanar image (EPI) gradient-echo pulse sequence (TR/TE = 475/30 ms, flip angle = 60º, 3 mm isotropic voxels, multiband factor = 8, interleaved).

Structural image processing was as follows: in FSL (Smith et al., 2004), alignment and averaging of multiple images, SUSAN noise reduction (Smith and Brady, 1997), anterior and posterior commissure (AC-PC) alignment, and non-linear registration to Montreal Neurological Institute (MNI) space; followed by brain segmentation and extraction in SPM12^1^. Time series images were processed as follows: motion correction via realignment, field map correction, despiking with AFNI’s 3dDespike (Jo et al., 2013), AC-PC alignment, coregistration to MNI space, smoothed with 6 mm FWHM kernel, and high pass filtered > 0.0078 Hz.

First level analyses were done in SPM12. To increase power, HED and LED images were combined. On an individual level, the BOLD (blood oxygen level dependent) response to food images was compared to the BOLD response to degraded images while covarying for motion (translation in x, y, z direction; pitch, roll, yaw movements). This was done for both the sham-deflation and the deflation condition.

Then, these food > degraded contrasts were compared on a group level to test for differences in BOLD response between the sham and deflation condition. To account for within-subject variance in the longitudinal data, these second level analyses were performed in the Sandwich Estimator (Guillaume et al., 2014) as implemented in FSL. Whole brain significance was determined through threshold free cluster enhancement (TFCE) (Smith and Nichols, 2009), with FWE-correction at *p* < 0.05. Clusters with ≥ 10 voxels are reported. TFCE settings were set to default (height = 2, extent = 0.5, cluster = 6). Age, age^2^ and current BMI were included in the model as nuisance variables.

Because of the small sample size, we also visually compared the BOLD response in the significant clusters to the BOLD response in a control cluster. This control cluster was defined as the BOLD response to the degraded images in the sham condition thresholded at *T* > 10 [bilateral clusters in the visual cortex [peak intensity at MNI 14, -94, 22 mm (442 voxels) and -16, -96, 14 mm (434 voxels)].

### Data analyses

Considering the small sample size, we used a non-parametric approach in form of permutation testing to test for differences in behavior (VAS motivation for food intake; guess of sham/deflation condition) between conditions. This was done with 2^6^ exhaustive permutations in R (R Core Team, 2017). For each subject, the real VAS difference between the conditions was multiplied by the random assignment of [-1,1], thereby permuting over condition. This was tested two-sided, with the *p*-value defined as the number of times the permuted outcome was lower (or higher for the opposite effect) than real outcome, divided by total number of permutations. Null distributions were visually checked for robustness.

To assess if participants could guess their condition better than chance, exhaustive samples (2*^n^*) were created of an n-sized vector with zeros and/or ones, representing an incorrect (zero) or correct (one) guess for each participant. Because of the small sample size, only a correct guess from all participants would have resulted in a condition-guess that significantly differed from chance.

## Results

### Behavioral results

Participants correctly identified the procedure as sham 60% of the time (3 out of 5 guessed correctly; missing for 1 participant) and as deflation 67% of the time (4 out of 6 guessed correctly). This is not different from chance (*p* = 0.50 and 0.66 for sham and deflation condition, respectively). The VAS on motivation for food intake differed between conditions for the question “Would you like to eat something sweet” [deflation (75.3 ± 11.6) > sham (54.9 ± 19.8), *p* = 0.03]. This did not survive FDR-correction (*p* = 0.28). The three other “would you like to eat something [salty/savory/fatty]” questions were not significant (*p* > 0.31); nor were the remaining five questions on feelings of hunger, fullness, and satiety (*p* > 0.22).

### Imaging results

**FIGURE 1:**
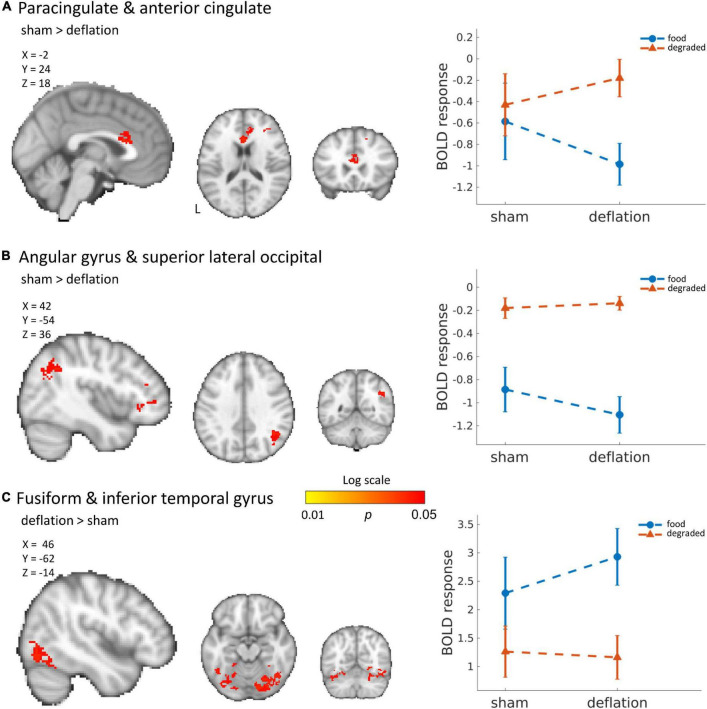
Significant deactivation in the deflation compared to sham condition for food images compared to degraded images in the cingulate cortex (A) and angular and superior lateral occipital cortex (B). Significantly increased activation to food images (compared to degraded images) in the deflation condition in the fusiform gyrus and inferior temporal gyrus (C). Line plots show mean activation in the respective significant clusters. Error bars represent SEM. Brains are in neurological orientation. See also Table 2. Unthresholded statistical maps have been uploaded to NeuroVault.org (Gorgolewski et al., 2015) and are available at https://neurovault.org/collections/KDJBAELV/.

Compared to sham-deflation condition, brain activity in response to food images in the (para)cingulate cortex, frontal cortex, angular gyrus, and superior lateral occipital cortex was decreased during deflation (Figures 1A,B and Table 2).

**TABLE 2:** Regions where activation in response to food images (compared to degraded images) differs between the sham (i.e., restricted) compared to deflated condition.

		MNI coordinates					
		
Region	Cognitive domain	X	Y	Z	*k*	z-stat*	*p*
**Sham > deflation**							
R angular, sup lat occipital	Information processing	42	−54	36	247	2.63	0.026
Bilateral ACC	Decision making	−2	24	18	221	2.31	0.026
R OFC, inf front gyrus, front pole	Behavioral control	42	32	−4	67	2.04	0.042
R frontal pole	Behavioral control	40	38	14	42	2.40	0.042
Bilateral paracingulate gyrus	Behavior regulation	2	44	6	20	2.17	0.042
R Frontal pole	Behavioral control	22	56	0	15	2.11	0.042
R superior frontal gyrus	Behavioral control	16	14	54	15	2.05	0.042
R superior frontal gyrus	Behavioral control	6	40	40	11	2.22	0.042
**Deflation > sham**							
R fusiform, inf temp gyrus	Higher-order visual proc	48	−56	−22	1,022	2.14	0.026
L fusiform, inf temp gyrus	Higher-order visual proc	−44	−60	−20	473	2.58	0.026
L sup lat occipital cortex	Higher-order visual proc	−28	−78	18	82	2.20	0.038
L occ fusiform gyrus	Higher-order visual proc	−18	−84	−14	22	2.00	0.038
L lingual gyrus	Visual processing	−2	−86	−4	13	2.26	0.038
L lingual gyrus	Visual processing	−14	−88	−10	12	2.02	0.038

*TFCE statistics are commonly high, which is why we report the z-statistic despite the small sample size. TFCE statistics ranged from 5,396 to 5,909 for Sham > Deflation, and 5,115–5,527 for Deflation > Sham. Unthresholded maps of both TFCE and z-statistics are available at https://neurovault.org/collections/KDJBAELV/. k, number of voxels in the significant cluster; p, TFCE-based whole-brain FWE-corrected p-value; MNI coordinates represent the peak-voxel location; R, right; L, left; sup, superior; lat, lateral; ACC, anterior cingulate cortex; inf, inferior; OFC, orbitofrontal cortex; front, frontal; temp, temporal cortex; occ, occipital; proc, processing.

Increased activation to food images during the deflation-condition was seen in the fusiform gyrus, inferior temporal gyrus, lingual gyrus, and superior lateral occipital cortex (Figure 1C and Table 2).

**FIGURE 2:**
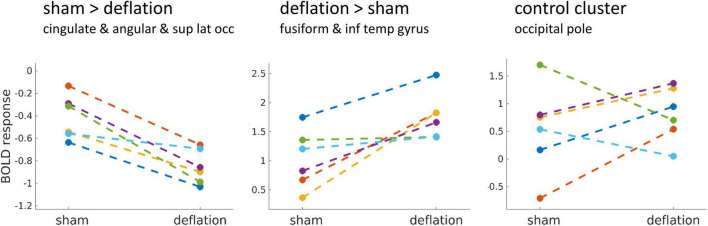
Mean brain activation in the significant clusters (left, middle) compared to a visual control cluster (right) during food image presentation minus brain activation during the degraded image presentation, for the sham and deflation condition. Each line is one participant. The control cluster was defined as the BOLD response to the degraded images in the sham condition thresholded at *T* > 10 and included bilateral clusters in the occipital pole (region of foveal vision processing). Sup, superior; lat, lateral; occ, occipital; inf, inferior; temp, temporal.

Change in activation between sham and deflation condition was similar for all individuals (Figure 2). This change was indeed specific to the significant clusters and did not reflect global interindividual differences as the change in activation in the occipital pole (peak activation to degraded images) differed between individuals (Figure 2).

## Discussion

Results from this small pilot study suggest that restriction caused by LAGB has an effect on several brain regions. Compared to sham-deflation (i.e., maintaining restriction) of the band, deflation (i.e., decreasing restriction) was associated with decreased activation in regions associated with information processing, behavior regulation, and behavioral control. Increased activation during deflation was seen in regions of (higher order) visual processing.

Our findings are in agreement with studies that report increased activation in behavioral control regions after bariatric surgery (Bruce et al., 2012; Goldman et al., 2013; Holsen et al., 2018; Zoon et al., 2018; Baboumian et al., 2019; Bach et al., 2021; Koenis et al., 2021), although some studies report a post-surgical decrease in the dorsolateral PFC (Ochner et al., 2012b), or decreased activity of the angular gyrus after weight loss intervention (Murdaugh et al., 2012). Activation of the visual cortex is also associated with food picture viewing (van der Laan et al., 2011; Tang et al., 2012; van Meer et al., 2015; Bach et al., 2021), and activity in these regions decreases after weight loss interventions (Bruce et al., 2014; Baboumian et al., 2019). Lower activation in visual processing areas during sham-deflation and after surgery could be related to decreased salience to (appetitive) food when the stomach is restricted (Tang et al., 2012).

Interestingly, we did not find changes in striatal reward areas or the insula, regions that have often been reported in relation to obesity and changes after bariatric surgery or behavioral weight loss intervention (Bruce et al., 2012; Murdaugh et al., 2012; Nock et al., 2012; Ochner et al., 2012a,b; Faulconbridge et al., 2016; Holsen et al., 2018). Possibly, changes in activity of reward regions are more related to the process of weight loss and habit formation rather than a direct mechanical effect. Alternatively, individual variance is higher in post-surgical changes in reward regions. In addition, most of these studies used preselected ROIs that did not reach whole brain significance.

Our findings suggest that post-surgical changes in brain responses to food pictures may in part directly be related to mechanical changes to the stomach. Possibly, some neuronal adaptation may occur early after surgery and underlie future changes in food craving and preference that support sustained post-surgical weight-loss. This may explain why post-intervention brain changes do not seem to be related to weight loss (Murdaugh et al., 2012; Koenis et al., 2021). Another example is the correlation between increased frontal and decreased visual cortex activity in response to food pictures and increased post-surgery GLP-1 (Baboumian et al., 2019). [GLP-1 is a satiety signal which increases early after surgery, but whose increase is not related to % TWL (Hutch and Sandoval, 2017)]. On the other hand, in a non-weight loss sample, increased frontal activity has been related to better regulation of craving and dietary restraint (DelParigi et al., 2007; Hollmann et al., 2012). Taken together, this suggests there may be multiple mechanisms at play.

Although studies on different bariatric surgery types report similar post-surgical changes, neuronal mechanisms may differ among surgery types: Faulconbridge found a decrease in ventral tegmental area (involved in reward processing) activity in response to food post-RYGB, but not in participants who underwent SG (Faulconbridge et al., 2016). Baboumian reports a stronger dorsolateral PFC increase post-RYGB compared to post-SG (Baboumian et al., 2019). In addition, brain changes may also differ between diet-intervention and LAGB: Bruce et al. (2014) report that decreases in the occipital cortex, among others, were larger post-LAGB compared to post-diet. Thus, the current results may not be generalizable to other types of bariatric surgery or to weight loss interventions.

Due to the pilot approach of this small study, there are several limitations to take into account when interpreting our results. We only included females; results may not generalize to males individuals as previous studies have demonstrated that women respond differently to visual food images (Chao et al., 2017). Participants all achieved successful weight loss at various time points after surgery. Mechanisms of neuronal adaptation to stomach restriction may differ in participants that do not achieve 45% TWL after LAGB. Last, our results are based on almost acute changes in stomach restrictions, and therefor do not allow for any extrapolated interpretation what this means to neuronal function several hours later.

In conclusion, our study provides additional evidence that surgical intervention may affect change in neuronal activation independent of weight loss, possibly via activation of mechanical baroreceptors in the area of gastric cardia and fundus during restriction of the band. Future studies could examine the neuronal associations of mechanical restriction in individuals with successful compared to unsuccessful weight loss following LAGB to discover mechanisms of successful weight loss. Other future directions include neuronal associations of mechanical restriction during the early adjustment phase in an effort to determine whether activation patterns related to mechanical restriction could be used to predict LAGB outcomes.

## Data availability statement

Unthresholded statistical maps are uploaded to NeuroVault.org (Gorgolewski et al., 2015) and are available at https://neurovault.org/collections/KDJBAELV/. More detailed information is not publicly available due to restrictions imposed by the administering institution and privacy of the participants. The authors will share them by request from any qualified investigator after completion of a data sharing agreement.

## Ethics statement

The studies involving human participants were reviewed and approved by the Hartford Hospital Institutional Review Board. The patients/participants provided their written informed consent to participate in this study.

## Author contributions

MK and JN analyzed the data and drafted the manuscript. BA acquired funding, designed the study, and coordinated data acquisition. AS, DT, and PP were involved in data acquisition. MS helped with statistical analyses and interpretation of the results. TM, AV, and MD involved in interpreting the results and revising the manuscript. GP contributed to the design of the study and revision of the manuscript. All authors approved the final submitted version.

## Footnotes

1
https://www.fil.ion.ucl.ac.uk/spm/software/spm12/
